# Impact of Surgeon Volume on Perioperative Complications and Survival to Total Hip Arthroplasty Following Femoral Head Core Decompression

**DOI:** 10.5435/JAAOSGlobal-D-24-00153

**Published:** 2024-11-19

**Authors:** Joshua G. Sanchez, Will M. Jiang, Meera M. Dhodapkar, Zachary J. Radford, Anthony E. Seddio, Mengnai Li, Daniel Wiznia, Jonathan N. Grauer

**Affiliations:** From the Yale Department of Orthopaedics and Rehabilitation, New Haven, CT.

## Abstract

**Introduction:**

Core decompression is a minimally invasive procedure considered in the treatment of early-stage femoral head osteonecrosis. This procedure is theorized to relieve vascular pressure and promote angiogenesis. Although a less invasive procedure, there are considerations related to learning curve, technique variations, etc. that may affect postoperative complications and longer term adverse events.

**Methods:**

Adult patients who underwent core decompression with a diagnosis of femoral head osteonecrosis were identified in the 2010-Q3 2021 PearlDiver M157 database. Core decompression surgeon volumes over the entire study period were assessed and divided into ranges: low (<5 procedures), medium (5 ≤ x ≤ 19 cases), and high (>19 cases) volumes. A 1:1:1 match based on age, sex, and Elixhauser Comorbidity Index was completed. Rates of 90-day complications were compared with univariable and multivariable analyses. Survival to total hip arthroplasty (THA) and to subsequent hip fracture at both 2 and 5 years were compared by Kaplan-Meier survival analysis.

**Results:**

The low-volume, medium-volume, and high-volume surgeon groups made up 87.5%, 11.9%, and 0.2% of core decompression volume, respectively. This was indicative of 6333 patients undergoing core decompression, and matching based on the surgeon-volume category led to 486 patients per group. No statistically significant differences were observed in shorter term complications and survival to THA or hip fracture at 2 or 5 years.

**Conclusion:**

Core decompression is a treatment option often considered for early-stage osteonecrosis. Critically, no differences were found in rates of any assessed complications between the surgeon-volume matched cohorts. These findings suggest that core decompression is a relatively safe procedure for surgeons of varying volume with this technique. Furthermore, this study suggests that higher volume surgeons are not conducting the procedure with expanded indications that might result in greater rates of conversion to THA.

Osteonecrosis of the femoral head may result from impaired blood flow.^1,2^ In certain cases, core decompression may be considered as a joint-preserving surgery to treat early-stage osteonecrosis of the femoral head.^3-7^ Core decompression involves drilling channels from the femoral neck to head to relieve vascular pressure and potentially improve blood flow and promote angiogenesis.^7,8^ The main goal of the procedure is to prevent additional progression of osteonecrosis and limit or delay the requirement of total hip arthroplasty (THA), which is the most definitive treatment of osteonecrosis of the femoral head.^7,9^

Core decompression is a safe and effective procedure, with a previous systematic review and meta-analysis by Hua et al^10^ finding an overall complication rate of 4.9% and success rate (defined as Harris hip score ≥70, no additional THA surgery required, no radiographic progression) of 65%. Furthermore, recent studies from Ng et al that used large, national administrative databases demonstrated an increasing incidence of femoral head osteonecrosis and the subsequent utilization of core decompression, especially in younger patients, from 2010 to 2020.^11,12^

Although core decompression has been demonstrated to be a safe procedure, it is still important to characterize preoperative factors that may increase the risk of postoperative complications. A previous study within a large, national (United States) database by Garcia-Lopez et al^13^ demonstrated that increased age is a critical patient-level preoperative factor that was the only analyzed variable predictive of conversion to THA. However, whether surgeon-specific factors such as surgeon volume for core decompression influence postoperative outcomes after this procedure has yet to be described. Like other procedures, surgeon experience is an important consideration because the learning curve may affect providers without notable previous experience, and the providers who do have notable experience may consider the procedure for patients with expanded indications.

As such, this study aimed to characterize the correlation of core decompression surgeon volume before the index procedure to rates of 90-day complications and 5-year conversion to THA or hip fracture in a large, national administrative database. We hypothesized that increased surgeon volume would be associated with lower rates of postoperative adverse events.

## Methods

### Patient Population

This retrospective study used the 2010-October 2021 M157 PearlDiver Mariner Patient Claims Database (PearlDiver Technologies). PearlDiver M157 is a large database that contains records from approximately 157 million orthopaedic patients and is well established for use in orthopaedic studies.^14-17^ Because the data are output in deidentified and aggregated nature, our institutional review board deemed studies using this data set exempt from review.

Patients with an osteonecrosis diagnosis of the femoral head were identified using International Classification of Diseases, 9th and 10th Revision (ICD-9 and ICD-10), coding. Patients who underwent a core decompression with an osteonecrosis ICD-9/10 diagnostic code on the same day as the procedure were then identified using Current Procedural Terminology (CPT) codes CPT-27299, CPT-27071, CPT-26992, and CPT-S2325.^13,18^

### Core Decompression Surgeon Volume

The distribution of core decompression surgeon volumes was assessed using the “PROVIDERREPORT” function directly within the PearlDiver interface. This function provides a report that describes the number of previous core decompressions for each of the selected providers within the PearlDiver database.

A histogram displaying the number of providers within the specified category of core decompression volume was then created based on the data gathered through the “PROVIDERREPORT” function. Based on this distribution, 3 categories of surgeon volume were defined, low volume (<5 procedures), medium volume (5 to 19 procedures), and high volume (>19 procedures), over the entire study period. The high-volume definition was chosen to allow for sufficient privacy for surgeons within the cohort.

### 90-Day Outcomes After Core Decompression Based on Surgeon-Volume Category

Patients who underwent core decompression by surgeons in the previously defined low-volume, medium-volume, or high-volume groups were identified and categorized. Patients in each cohort were matched 1:1:1 based on age, sex, and Elixhauser Comorbidity Index (ECI) using methods previously described.^14^

90-day postoperative complications were then identified based on ICD coding using methods previously described.^14,19^ Serious adverse events were defined as at least 1 occurrence of surgical site infection, cardiac arrest, myocardial infarction, sepsis, and venous thromboembolism (pulmonary embolism or deep vein thrombosis). Minor adverse events were defined as at least 1 occurrence of transfusion event, wound dehiscence, hematoma, acute kidney injury, urinary tract infection, or pneumonia. Any adverse events represented the presence of at least 1 occurrence of either a serious adverse event or a minor adverse event.

90-day readmissions were also identified using the “ADMISSIONS” code within the PearlDiver interface as previously described.^19-21^ Readmissions were not included in the any adverse event analysis.

### Survival to Subsequent Hip Fracture or Conversion to Total Hip Arthroplasty Within 2 and 5 Years

Patients with a laterality-specified osteonecrosis ICD-10 code on the day of the initial core decompression procedure were selected. Patients with a laterality-specified osteonecrosis ICD-10 code on the day of THA were also identified. Finally, those with a previous laterality-specified core decompression for osteonecrosis who converted to an ipsilateral THA within 2 or 5 years of core decompression were defined.

Patients within the matched cohorts with an ICD-9 or ICD-10 diagnostic code of a hip fracture within 2 years and 5 years after the index core decompression were also identified. Patients with closed femoral head/neck, intertrochanteric, or subtrochanteric fractures were included.

### Statistical Analysis

For patient demographics, age and ECI were compared using the unpaired 2-tailed Student *t*-test and patient sex was compared using the Pearson chi-squared test. To match patients within the 3 volume cohorts, the “MATCH” function was used within the PearlDiver interface. For comparison of all patient characteristics, significance was defined as *P* ≤ 0.05.

For 90-day complication analysis between the matched cohorts, univariable analysis was conducted with the Pearson chi-squared test or Fisher exact test where appropriate. Multivariable logistic regression, controlling for age, sex, and ECI, was then conducted to determine the odds ratios and 95% confidence intervals of shorter term complications for the specified cohort of patients with core decompression versus the matched high-volume cohort. Statistical significance was defined as *P* ≤ 0.0017 as calculated by Bonferroni correction.

Kaplan-Meier survival curves were constructed to compare 2-year and 5-year survival to subsequent ipsilateral THA and hip fracture between the matched study groups using methods previously described.^21^ A log-rank test was conducted to evaluate any statistically significant difference between the matched study groups. Statistical significance was defined as *P* ≤ 0.05.

All statistical analyses were conducted using PearlDiver Bellwether and GraphPad Prism v9.4.1 (GraphPad Software).

## Results

### Core Decompression Cohorts

**Figure 1:**
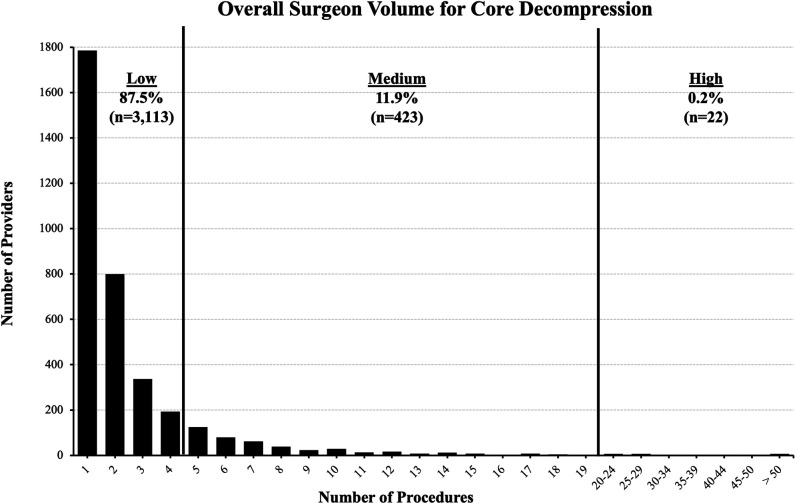
Chart demonstrating the overall surgeon volume of core decompression from 2010 to October 2021 in the study data set. The number of providers (y-axis) who have completed the specified number of core decompression procedures (x-axis) in this period. Black vertical lines signify the cutoff for the respective category of volume: low-volume surgeons = <5 procedures, medium-volume surgeons = 5 to 19 procedures, and high-volume surgeons >19 procedures. The number of providers and percentage of each group is also shown.

A total of 3557 providers performing core decompressions were identified. Of these, low-volume surgeons were 87.5% (n = 3,113), medium-volume surgeons were 11.9% (n = 423), and high-volume surgeons were 0.2% (n = 22) (Figure 1).

These providers performed core decompressions in a total of 6,333 patients. Of these patients, low-volume surgeons performed 3,664 (57.9%) of the surgeries, medium-volume surgeons performed 1,754 (27.7%) of the surgeries, and high-volume surgeons performed 694 (14.4%) of the surgeries (Supplementary Table 1, http://links.lww.com/JG9/A370). In the unmatched groups, patients in the high-volume surgeon cohort tended to be older (46.6 ± 15.0 vs. 45.0 ± 15.0 [medium-volume] vs. 45.0 ± 14.2 [low-volume], *P* = 0.001), more female (47.5% vs. 44.6% [medium-volume] vs. 41.9% [low-volume], *P* = 0.004), and sicker as measured through ECI (4.6 ± 3.9 vs. 4.5 ± 3.8 [medium-volume] vs. 4.2 ± 3.6 [low-volume]) (Supplementary Table 1, http://links.lww.com/JG9/A370).

After a successful 1:1:1 match, 486 patients remained in each group. No remaining differences were observed in age, sex, or ECI after the matching process (Supplementary Table 1, http://links.lww.com/JG9/A370).

### Shorter and Longer Term Complications After Core Decompression Based on Surgeon-Volume Group

The matched surgeon-volume cohorts demonstrated no statistically significant difference for any analyzed 90-day complication in the univariable (Table 1) or multivariable (Table 2) analysis after the Bonferroni correction. Readmission rates within 90 days were also not notable between the patient groups (Tables 1 and 2).

**Table 1:** Univariable Analyses of 90-Day Complications and Readmissions

Factor or Variable	Low Volume (n = 486)	Medium Volume (n = 486)	High Volume (n = 486)	*P* ^a^
Any adverse events	47 (9.7%)	35 (7.2%)	43 (8.9%)	0.375
Severe adverse events	17 (3.5%)	19 (4.0%)	22 (4.5%)	0.711
Surgical site infection	<11	<11	<11	0.049
Cardiac-related events	<11	<11	<11	0.605
Sepsis	<11	<11	<11	0.448
Venous thromboembolism	12 (2.5%)	<11	14 (2.9%)	0.430
Minor adverse events	37 (7.6%)	20 (4.1%)	28 (5.8%)	0.067
Bleeding-related events	12 (2.5%)	<11	<11	0.104
Wound dehiscence	<11	<11	<11	0.005
Acute kidney injury	<11	<11	<11	0.144
Urinary tract infection	17 (3.5%)	<11	<11	0.111
Pneumonia	<11	<11	<11	0.701
Readmissions	37 (7.6%)	44 (9.1%)	26 (5.3%)	0.083

a Bonferroni correction: *P* ≤ 0.0017.

**Table 2:** Multivariable Analyses (Controlling for Age, Sex, and Elixhauser Comorbidity Index) of 90-Day Complications and Readmissions

Factor or Variable	Low Volume OR (95% CI)	*P*	Medium Volume OR (95% CI)	*P* ^a^
Any adverse events	1.16 (0.74-1.81)	0.522	0.79 (0.49-1.27)	0.338
Severe adverse events	0.78 (0.39-1.49)	0.447	0.86 (0.45-1.61)	0.629
Surgical site infection	1.07 (0.13-8.91)	0.950	4.06 (1.01-27.00)	0.078
Cardiac-related events	0.35 (0.02-2.80)	0.372	0.66 (0.10-4.04)	0.655
Sepsis	2.17 (0.21-47.00)	0.527	1.00 (0.04-25.34)	1.000
Venous thromboembolism	0.92 (0.41-2.03)	0.838	0.56 (0.22-1.33)	0.200
Minor adverse events	1.41 (0.84-2.40)	0.196	0.70 (0.38-1.26)	0.232
Wound dehiscence	—	0.989	0.24 (0.04-0.98)	0.075
Bleeding-related events	2.18 (0.77-7.10)	0.158	1.00 (0.28-3.62)	1.000
Acute kidney injury	1.10 (0.33-3.59)	0.881	0.16 (0.01-0.96)	0.092
Urinary tract infection	2.37 (1.01-5.99)	0.054	1.13 (0.42-3.10)	0.804
Pneumonia	1.08 (0.37-3.20)	0.885	0.71 (0.21-2.25)	0.562
Readmissions	1.39 (0.82-2.40)	0.222	1.78 (1.10-2.97)	0.026

CI = confidence interval, OR = odds ratio

a Bonferroni correction: *P* ≤ 0.0017.

Results were underpowered and not reported.

**Figure 2:**
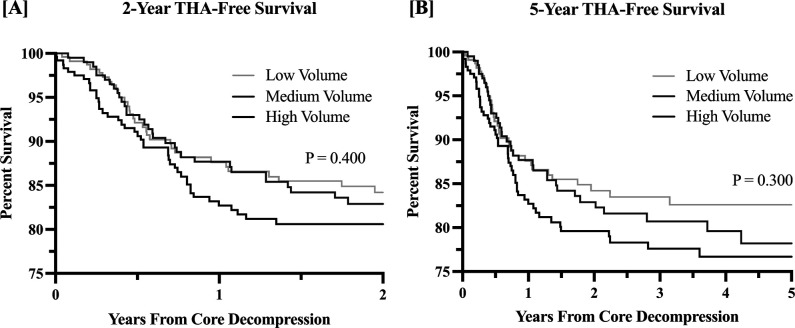
**A**, Graph demonstrating Kaplan-Meier survival analysis of time from core decompression to total hip arthroplasty (THA) within 2 years of the index procedure. **B**, Graph demonstrating Kaplan-Meier survival analysis of time from core decompression to THA within 5 years of the index procedure. Survival data of the 3 specified groups are shown for both.

**Figure 3:**
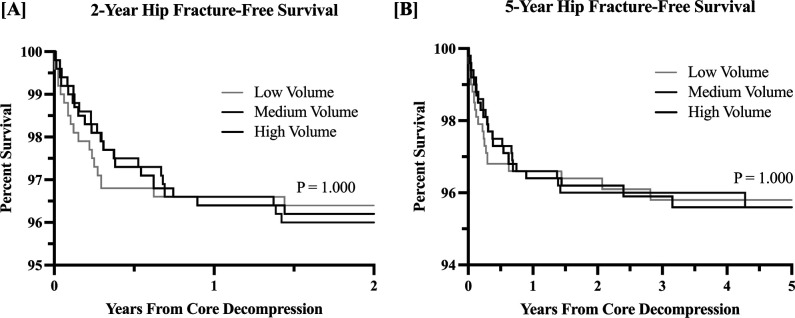
**A**, Graph demonstrating Kaplan-Meier survival analysis of time from core decompression to proximal hip fracture within 2 years of the index procedure. **B**, Graph demonstrating Kaplan-Meier survival analysis of time from core decompression to proximal hip fracture within 5 years of the index procedure. Survival data of the 3 specified groups are shown for both.

For the survival analysis, there were no differences in conversion to ipsilateral THA (Figure 2, A and B, respectively) or subsequent hip fracture within 2 or 5 years (Figure 3, A and B, respectively) between the matched study groups.

## Discussion

Core decompression of the femoral head may be offered to patients with early-stage osteonecrosis. Previous studies have focused on overall success rates of the procedure.^6,10,22^ Given that physician experience with core decompression may be varied, the correlation of surgeon experience with shorter and longer term complications after core decompression is of clinical interest.

The distribution of core decompression surgeon volumes suggests that most providers had performed relative low volumes of this procedure. Specifically, 87.5% of surgeons performing core decompressions were found to have performed less than 5 procedures over the entire study period. Meanwhile, only 0.2% of the providers had performed 20 or more procedures over the study period.

A total of 6314 core decompression patients were identified. Most patients (3664; 57.9%) underwent core decompression by a provider who performed less than 5 procedures from 2010 through 2021. Interestingly, the high-volume surgeon group performed surgeries on patients who were slightly older, were female, and had higher comorbidity burden. To put this in context, a previous core decompression study by Garcia-Lopez et al^13^ found older age to be a predictor in conversion of core decompression to THA.

To allow for direct comparison of patient outcomes, the different surgeon volume groups were matched based on age, sex, and ECI to mitigate confounding effects. Based on univariable and multivariable analyses, no differences were found in 90-day postoperative complications and readmissions. This is reassuring in light of the fact that differences in surgeon volume have been demonstrated to negatively affect outcomes after other common orthopaedic procedures, such as reverse total shoulder arthroplasty^23^ and total joint arthroplasty (hip and knee).^24^ The decreased invasiveness and baseline complications of core decompression^10^ may have contributed to the lack of differences found between volume categories within this study.

Regarding rates of 2-year and 5-year ipsilateral hip fracture and conversion of core decompression to THA, no differences were found based on surgeon-volume group. These findings are further reassuring that the outcomes of surgeons with lower volumes are analogous to those with higher volumes. Taken in the opposite direction, these findings are reassuring to those with higher volume core decompression practices because they are not having higher failures that might be associated with stretched surgical indications. Noteworthy, even high-volume surgeons completed relatively few core decompression procedures because an average of 2 procedures per year satisfied the cutoff used in this study.

There are several limitations of this study. First, as an administrative database analysis, this study is limited by coding quality and patient-level characteristics are unavailable in this database. In addition, the exclusion of patients without laterality-coded osteonecrosis may have led to underreporting in the ipsilateral revision analysis. Second, osteonecrosis stage and size/location of the lesion were unable to be analyzed in this database. Therefore, this study could not control patient-level differences in osteonecrosis disease severity among the study groups. Finally, provider experience was defined by the number of core decompressions performed during the entire study period. Training information, years of surgical experience, and other experience metrics could not be obtained from the database. We could not obtain information on surgeon's techniques regarding the size of drill, how many passages, and whether fluoro or navigation technology was used. Finally, higher procedure volume cutoff values were not completed because of potential privacy concerns for the very limited number of very high-volume surgeons. One additional limitation is that we were not able to extract data to compare/match patients who were diagnosed with early-stage osteonecrosis but did not undergo core decompression regarding their outcomes (including survival analysis).

## Conclusion

Core decompression is a joint-sparing procedure often used to treat patients with early-stage osteonecrosis. Although most of the core decompressions were performed by surgeons with less than previous 5 cases over the study period, no difference in postoperative adverse events, longer term hip fracture, or conversion to THA were found when comparing low-volume, medium-volume, and high-volume providers as defined by the range of cases identified in the data set.

## Supplementary Material
