# Supplementary material for: Impact of Surgeon Volume on Perioperative Complications and Survival to Total Hip Arthroplasty Following Femoral Head Core Decompression
Source: J Am Acad Orthop Surg Glob Res Rev. 2024 Nov 19;8(11):e24.00153. doi: 10.5435/JAAOSGlobal-D-24-00153 (PMC11578193; doi:10.5435/JAAOSGlobal-D-24-00153)
Supplement: Supplementary file 1 [file jagrr-8-e24.00153-s001.docx]

**Supplementary Table 1.** Demographics of core decompression patients. Groups defined by surgeon volume: low-volume (< 5 procedures), medium-volume (5 to 19 procedures), and high-volume (> 19 procedures). A match controlling for age, sex, and ECI is shown.

|  | **Non-Matched Groups** | | |  | **Matched Groups (1:1:1)** | | |  |
| --- | --- | --- | --- | --- | --- | --- | --- | --- |
|  | **Low Volume** | **Medium Volume** | **High Volume** | **P-value** | **Low Volume** | **Medium Volume** | **High Volume** | **P-value** |
| **Total** | 3,664 | 1,754 | 915 |  | 486 | 486 | 486 |  |
| **Age (mean**$\boldsymbol{\pm}$ **SD)** | 45.0 $\boldsymbol{\pm}$ 14.2 | 45.0 $\boldsymbol{\pm}$ 15.0 | 46.6 $\boldsymbol{\pm}$ 15.0 | **0.001** | 45.4 $\boldsymbol{\pm}$ 11.8 | 45.4 $\boldsymbol{\pm}$ 11.8 | 45.4 $\boldsymbol{\pm}$ 11.8 | 0.849 |
| **Sex** |  |  |  | **0.004** |  |  |  | 1.000 |
| **Female** | 1,534  (41.9%) | 782  (44.6%) | 435  (47.5%) |  | 190  (39.1%) | 190  (39.1%) | 190  (39.1%) |  |
| **Male** | 2,130  (58.1%) | 972  (55.4%) | 480  (52.5%) |  | 296  (61.0%) | 296  (61.0%) | 296  (61.0%) |  |
| **ECI (mean**$\boldsymbol{\pm}$ **SD)** | 4.2 $\boldsymbol{\pm}$ 3.6 | 4.5 $\boldsymbol{\pm}$ 3.8 | 4.6 $\boldsymbol{\pm}$ 3.9 | **0.006** | 3.2 $\boldsymbol{\pm}$ 2.6 | 3.2 $\boldsymbol{\pm}$ 2.6 | 3.2 $\boldsymbol{\pm}$ 2.6 | 0.930 |

ECI = Elixhauser Comorbidity Index, SD = Standard Deviation
